# Review of attacks on health care facilities in six conflicts of the past three decades

**DOI:** 10.1186/s13031-018-0152-2

**Published:** 2018-05-02

**Authors:** Carolyn Briody, Leonard Rubenstein, Les Roberts, Eamon Penney, William Keenan, Jeffrey Horbar

**Affiliations:** 1 Mailman School of Public Health Columbia University, New York, NY USA; 2 Johns Hopkins Bloomberg School of Public Health, Baltimore, USA; 3 School of Medicine, St. Louis University, St. Louis, USA; 4 Larner College of Medicine, University of Vermont, and Vermont Oxford Network, Burlington, USA

**Keywords:** Attacks, Health care, Facilities, Syria, Yemen, Surveillance, Conflict

## Abstract

**Background:**

In the ongoing conflicts of Syria and Yemen, there have been widespread reports of attacks on health care facilities and personnel. Tabulated evidence does suggest hospital bombings in Syria and Yemen are far higher than reported in other conflicts but it is unclear if this is a reporting artefact.

**Objective:**

This article examines attacks on health care facilities in conflicts in six middle- to high- income countries that have occurred over the past three decades to try and determine if attacks have become more common, and to assess the different methods used to collect data on attacks. The six conflicts reviewed are Yemen (2015-Present), Syria (2011- Present), Iraq (2003–2011), Chechnya (1999–2000), Kosovo (1998–1999), and Bosnia and Herzegovina (1992–1995).

**Methods:**

We attempted to get the highest quality source(s) with summary data of the number of facilities attacked for each of the conflicts. The only conflict that did not have summary data was the conflict in Iraq. In this case, we tallied individual reported events of attacks on health care.

**Results:**

Physicians for Human Rights (PHR) reported attacks on 315 facilities (4.38 per month) in Syria over a 7-year period, while the Monitoring Violence against Health Care (MVH) tool launched later by the World Health Organization (WHO) Turkey Health Cluster reported attacks on 135 facilities (9.64 per month) over a 14-month period. Yemen had a reported 93 attacks (4.65 per month), Iraq 12 (0.12 per month), Chechnya > 24 (2.4 per month), Kosovo > 100 (6.67 per month), and Bosnia 21 (0.41 per month). Methodologies to collect data, and definitions of both facilities and attacks varied widely across sources.

**Conclusion:**

The number of reported facilities attacked is by far the greatest in Syria, suggesting that this phenomenon has increased compared to earlier conflicts. However, data on attacks of facilities was incomplete for all of the conflicts examined, methodologies varied widely, and in some cases, attacks were not defined at all. A global, standardized system that allows multiple reporting routes with different levels of confirmation, as seen in Syria, would likely allow for a more reliable and reproducible documentation system, and potentially, an increase in accountability.

## Background

### Introduction

The Geneva Conventions and the Rome Statute demand protections for health facilities and health care providers. Yet breaches of these protections have been reported by authoritative sources in Yemen and Syria without eliciting an effective response from the international community.

### Objective

We attempt to determine whether attacks on health facilities in the current conflicts in Yemen (2015-Present) and Syria (2011- Present) have become more commonplace compared to earlier conflicts in Iraq (2003–2011), Chechnya (1999–2000), Kosovo (1998–1999) and Bosnia and Herzegovina (1992–1995), and to gain lessons revealed by the different methods utilized for monitoring attacks in these conflicts.

### Methods

Four conflicts considered most comparable to Syria and Yemen were selected in middle- to upper-income countries where reporting of attacks was likely to occur. Initially we conducted a search in MEDLINE, PubMed, ProQuest, Google Scholar and Google using combinations of the following keywords: hospital(s), clinic, facility, health care system, private health care, number, doctors, nurses, attack(ed), bombing, killed, Bosnia, Bosnia and Herzegovina, Iraq, Yemen, Syria, Kosovo, Chechnya, 2nd Chechen War. Custom date ranges on Google and Google Scholar were used to exclude events and reports that were published before or after the conflicts. The data identified included primary and secondary sources consisting of newspaper articles, peer-reviewed journal articles, and open- source platforms. Secondary sources were identified as references in primary documents. In all conflicts except Iraq, summary or review articles of attacks on facilities existed and created a more complete (in terms of number of attacks) record than the sum of all identified reports. We attempted to use the best summary data of attacks on health care for each of the conflicts, sometimes combining information from different summaries. Best source is defined as a credible authority, such as the WHO, and broadest coverage in terms of time and space. In Iraq, we tallied individual reported events of attacks on health care. Summary data in the other conflicts came from Physicians for Human Rights (PHR), the World Health Organization (WHO), the Bosnian Ministry of Health, the World Bank and Unicef.

The time range identified for each of the conflicts is based on the source’s period of review. Because there is no summary source for Iraq, the duration that was used for the search and tally is the official period of American occupation (2003–11).

An attack on a health care facility was a priori defined as any form of physical violence or obstruction that interferes with the accessibility and delivery of health care, including all types of structures that provide health care and transport to medical facilities. This definition was used for the conflict in Iraq because we tallied individual events of attacks from newspaper and organization accounts. However, the concept and definition of an attack on a health care facility varied between the other summary sources. The source data for the conflicts in Bosnia and Herzegovina and in Yemen had narrow definitions and primarily included reports of attacks on hospitals. The population-based surveys undertaken by Physicians for Human Rights examining the conflicts in Chechnya and Kosovo defined an attack as an act of intentional destruction to a facility or clinic. For the conflict in Syria, Physicians for Human Rights defines an attack as a “violent assault upon a facility resulting in any destruction, damage or loss of the facility’s function, equipment or medical supplies [1].” The WHO Turkey Health Cluster does not explicitly define what they consider an attack, but the authors, “describe the use of this tool to produce primary data for attacks on health-care facilities, ambulances, health workers, and patients.” All of the incidents subsequently reported in the article are attacks on health facilities [2]. The source data from WHO and Unicef for Yemen do not define what they consider an attack on a health care facility.

While these definitions vary in many aspects, all consider an attack as a violent event that interferes with health care delivery, with the main difference relating to the inclusion criteria for what constitutes a facility. However, it is possible and likely that researchers attempted to assess the primary type of facility that was targeted during that conflict. For example, clinics were specifically targeted as Serbs systematically destroyed villages where Kosovar Albanians lived [3].

Efforts were made to estimate the total number of facilities in each location at the start of the conflict so that rates of attacks could be recorded. This proved very difficult because of contradictory estimates (e.g. Chechnya), out-of-date information or lack of private facility documentation (e.g. attack reports included events affecting both public and private facilities in Syria, but little information could be found regarding estimates of the number of private facilities in Syria.)

In the initial results, we report attacks using the language, facility or hospital, utilized by the source. For simplification and consistency thereafter, the term “facilities” is used in summary tables and discussion.

International law requires steps to avoid harm to medical personnel and facilities as much as possible [4, 5]. Indiscriminate bombing and attacks that affect health care still violate international law, and thus we include data about attacks regardless if intentionality is known or not. However, where intentionality was reported by authors as part of the attack definition or the data collected, it is presented in the discussion section. All attacks are given equal weight, regardless of the size of facility in our summaries.

## Results

Physicians for Human Rights reported 465 attacks on 315 different medical facilities from March 2011 to March 2017 in Syria. The method for collecting data is done through a three step process, 1) open source searches on the internet, 2) triangulation with additional open source reports, and 3) corroboration with satellite images and partners in-country [1, 6]. The Monitoring Violence against Health Care (MVH) network launched by the WHO Turkey health cluster reported 402 attacks, with attacks on 135 different facilities, over the period from November 2015–December 2016 in Syria. The method to collect and verify data requires an internal health cluster partner to send an alert with information of an attack to a WhatsApp group, and a second external partner then verifies the attack by physically visiting the location. [2]. In Yemen, the WHO reported that between March 2015 and August 2016, 102 health care facilities have been partially or totally damaged, and Unicef has orally verified but not published reports on 93 attacks on hospitals from March 2015 to December 2016 [7, 8]. Neither of these sources included methodology information on how data was collected for these attacks.

In Iraq between March 2003 and December 2011, an estimated 12 health facilities were bombed, stormed, or attacked by American forces and suicide bombers [9–21]. These sources of data were all events captured by the media. The methodologies to verify this information are not defined in the published articles. In Bosnia and Herzegovina, initial sources pointed to 18 hospitals attacked and were corroborated by reports from a Bosnian Ministry of Health official and the World Bank, which stated that 30% of hospitals were destroyed during the conflict [22–28]. There were a reported 70 hospitals in Bosnia and Herzegovina before the start of the conflict, which places the estimate of attacks at 21 hospitals [29]. The Bosnian Ministry of Health official and the World Bank did not cite the methodology for determining the estimate of 30% destruction of hospitals, but it can be assumed that this information was well known given the small number of reported hospitals in the country.

Population-based surveys were conducted by Physician for Human Rights in partnership with the Program on Forced Migration at Columbia University in Kosovo and Chechnya. In Kosovo, between February 1998 and June 1999, PHR confirmed the “intentional destruction” of at least 100 clinics and 24 facilities in Chechnya between August 1999 and May 2000 [3, 30]. The survey of Kosovar refugees utilized a modified form of systematic probability sampling in refugee camps [3]. Respondents in the Chechen survey were randomly selected from refugee camp registrations. In both, interviewers asked respondents if they had seen medical facilities that were destroyed, who the perpetrator was, date of the attack, name and location of facility [3, 30].

Table 1 below suggests that Syria has the greatest number of reported facilities attacked compared to all other conflicts. Yemen and Kosovo have similarly reported high numbers, followed by a distinct decrease in the 2nd Chechen war and Bosnia. Iraq has the least number of reported attacks.

**Table 1:** Total number of facilities attacked

Conflict	Number of reported facilities attacked
Syria	315/135^a^
Yemen	93
Iraq	12
2nd Chechen War	> 24
Kosovo	> 100
Bosnia	21

^a^PHR reported #/MVH reported # in 20% of time

The number of facilities attacked per month is presented in Table 2 with the number of facilities attacked divided by the length of the review period. When using the MVH findings, Syria has the greatest number of facilities attacked per month at 9.64 per month. Kosovo has the second highest number of facilities attacked per month at 6.67 per month. Yemen follows at 4.65 per month. The Physicians for Human Rights Syria figure for Syria 4.38 facilities attacked per month.

**Table 2:** Facility attacks per month

Conflict	Length of source’s period of review (months)	Facilities attacked per month
Syria	72/14^a^	4.38/9.64^a^
Yemen	20	4.65
Iraq	104	0.12
2nd Chechen War	10	2.4
Kosovo	15	6.67
Bosnia	44	0.41

^a^PHR reported first, MVH second

## Discussion

There appears to be a tendency in the press and among scholars to report human rights violations, such as executions of children or killing of UN Peacekeepers, as numbers, not as rates. This implies that each such event is unacceptable and noteworthy on its own. We consider an attack on a health care facility such an event. This makes the comparison of conflict-related attacks on health facilities meaningful, even with countries of vastly different sizes and unknown denominators of legally protected health care sites.

The data uncovered from the literature review may be incomplete and not fully accurate in some cases. Sources of information across conflicts both defined and collected data on attacks in different ways. While likely incomplete, these conflicts were in highly developed countries and experienced considerable press scrutiny, and thus likely constitute a significant fraction of events. Undoubtedly, attacks on health care facilities during war are not a new phenomenon. Yet, this research suggests that the conflict in Syria has experienced far more events compared to the other conflicts examined, even though Iraq and Yemen have larger populations, and the Iraqi conflict lasted longer. Also, given conflicting reports and the existence of private health facilities in places like Syria, we found it nearly impossible to determine the denominator of facilities at risk and therefore we do not report rates of attacks.

Syria provides the only context in which two methodologies can be compared. It is not possible to draw a conclusion about the sensitivity of the two methodologies without access to their databases to examine individual events. It is also not feasible to compare the raw numbers or the number of facilities attacked per month because they cover different time periods. However, it is worth nothing that in 2016, Physicians for Human Rights reported a total of 108 attacks on facilities, and MVH reported 158 attacks from November 2015–December 2016 [1, 2]. While the time period is not exactly the same, it appears that MVH captured a greater number of reported events during 2016. Given the provided data, it cannot be determined if the difference in reported numbers is due to different sensitives of the systems, different geographic spread of captured events, different verification processes or a combination of factors.

The data in Yemen is also extremely troubling and suggests that attacks on health care occurred regularly during the majority of 2015 and 2016, but without clear knowledge of the methodologies used for collecting data on attacks, it is impossible to assess the monitoring mechanisms utilized.

It was not possible for the authors to clearly distinguish intentionality of attacks due to varying methodologies. Some sources of data used intentionality as inclusion criteria, like for the conflicts in Chechnya and Kosovo. But for others, intentionality is not included in the definition of an attack on health care, making it difficult to assess this factor’s relationship with the apparent increase in attacks. For many conflicts it is not clear if destruction of facilities is a byproduct of the failure to take precautions required by Geneva Conventions or if attacks were a deliberate strategy. Moreover, reports do not allow perfect insight into the total number of attacks. For example, the United States and allies likely dropped half a million tons of bombs in Iraq by 2005, potentially masking the extent to which those forces avoided health facilities [31]. The reports of attacks on health care in Yemen are characterized by this uncertainty of intentionality [6, 7].

In Kosovo, the intentionality of destroying health facilities is well understood. Serbs were targeting Kosovar Albanians and systematically destroyed towns where this population lived, burning clinics as well as house and schools. They did not destroy hospitals [3].

In Syria, the intentionality of many attacks is clear through evidence presented by Physicians for Human Rights. They report that 24% of the facilities attacked have been attacked more than once, 60% of all attacks were carried out with discriminate weapons (mortars, missiles, rockets, etc.), and 31% of facilities attacked were located in isolated or sparsely populated areas away from other buildings [1]. During the siege of Aleppo in July of 2016, the four remaining facilities were bombed on the same day [32]. Events like this are only plausibly explained by intentionality.

There are many limitations to be considered with this analysis. The conflict in Bosnia, and to a lesser degree in Kosovo and Chechnya, happened during a period when the internet was less effectively capturing public events. On the other hand, population-based surveys like those conducted in Chechnya and Kosovo are not seen in other conflicts. Therefore, the data found for facilities attacked may or may not be fully complete. The estimates for Syria and Yemen also are not completely up to date, as we decided to use data from Physicians for Human Rights and Unicef reported in spring 2017 and 2016, respectively. Furthermore, the varied definitions of an attack and methodologies indicate that the same type of event has a different probability of being captured in different countries, making the comparison of data across conflicts challenging. Our analysis and conclusion are based off these rough estimates.

We recognize that the number of facilities attacked does not address attacks on health personnel. In a study conducted of occupied Iraq by Burnham et al. in 2012, the violent death rate of physicians in Baghdad peaked at 47.6/1000/year and remained constant outside of Baghdad at 25.3/1000/year during the conflict, at least twice that of the general population [33]. It is not conclusive whether the increased rate of murders of medical professionals during this time is a deliberate attack on health care or due to other considerations such as status and class of physicians in Iraq. Another estimate made in 2007 by the Iraq Medical Association reported that 2000 doctors had been killed since the start of the 2003 invasion [34]. In Kosovo before the conflict, Albanian doctors were systematically persecuted by Serbs, and were subject to mass firings and elimination of medical training opportunities [3]. In the survey of the conflict, “PHR has documented the targeting and murder of at least three physicians in the fall of 1998 and targeted murders of at least nine others in the past several months” during the short conflict [3]. These examples highlight that the facility attack reports we analyzed are not sufficient to determine the overall level of violence inflicted on health care in these conflicts.

Finally, social medial and reporting mechanisms may naturally be improving with time, making surveillance of such events better. We choose the conflicts in Bosnia, Chechnya, and Kosovo because they had been intensively studied, which we hoped would help to correct for this early Internet age. But, this conscious selection excludes conflicts like those in the Central African Republic and South Sudan where attacks on medical facilities may be widespread, but monitoring is believed to be relatively incomplete.

## Conclusion and recommendation

While our study revealed that attacks on health care in conflicts are not a new phenomenon, and it is not easy to compare conflicts from 20 years ago with today, there appears to be a comparatively high number of attacks on hospitals in Syria. The figures in Yemen are also troubling, but the lack of defined methodologies prevents us from reaching a firm conclusion. It also cannot be determined if intentional attacks on health care have increased given the varying definitions of what constitutes an attack in the methodologies. We believe that Physicians for Human Rights and the Monitoring Violence against Health Care network have demonstrated that it is possible to implement a surveillance system to capture attacks on health care in a time of war. They have documented evidence of intentional and repeated patterns of attacks. This is invaluable because those who carry out the abuses cannot deny these acts at a later time. A global, standardized surveillance system that utilizes primary and secondary sources must be implemented in Yemen, and in other fragile contexts.

The World Health Assembly’s 2012 Resolution requires the World Health Organization to establish this global system for the collection and dissemination of data so that the conversation and evidence is expanded to include all other conflicts [35]. It is disappointing that the implementation of this system has been delayed several times, given its importance. However, after lessons that were learned from earlier iterations in Afghanistan and Syria, we note that the methodology for this surveillance system has now been finalized and is being rolled out. It will utilize a combination of primary and secondary sources for capturing and verifying events.

Regardless of whether Syria is an extremely egregious violation of International Humanitarian Law compared to past conflicts, or attacks of health facilities have been better documented in Syria than in past conflicts, it is discouraging that there has been impunity for these hundreds of incidents. The less clear image of health care attacks documented by WHO and UNICEF in Yemen suggest little hope in this setting for legal or political recourse for such violations. This hopelessness, and the difficulties in this review in establishing a typical level of collateral health care damage, highlights the logic and importance of the 2012 World Health Assembly demand that WHO establish a systematic monitoring process for health care attacks.

### This article is dedicated to Dr. Mohammad Wassim Maaz:

**Fig. 1:**
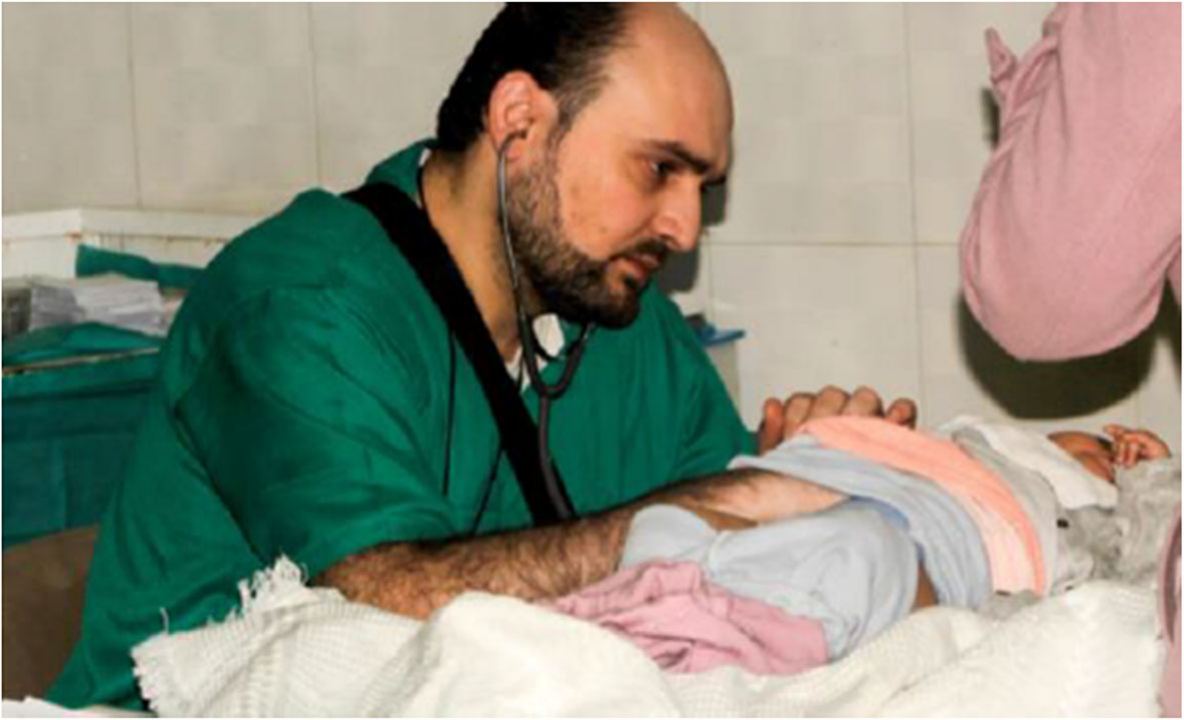
The Syrian pediatrician, Dr. Mohammad Wassim Maaz. Source: https://www.theguardian.com/world/2016/apr/29/mohammad-wassim-maaz-syrian-paediatrician-al-quds-airstrike

The Syrian pediatrician, Dr. Mohammad Wassim Maaz died in an airstrike on the al-Quds hospital in Aleppo, Syria in April 2016 which killed him, a dentist, three nurses and 22 civilians (Fig. 1). The Al-Quds hospital was supported by Médecins Sans Frontières and the International Committee of the Red Cross. A group of doctors still practicing in Aleppo commemorated him by saying, “We will always remember Dr. Maaz as the kindest and bravest of souls, whose devotion to treating the youngest victims of this war was unparalleled,” We dedicate our research to the memory of Dr. Mohammad Wassim Maaz and to the other physicians, nurses, and allied health professionals around the world who have died practicing in the face of humanitarian crises and war.

## Abbreviations

MSF: Doctors Without Borders
MVH: Monitoring Violence against Health Care
PHR: Physicians for Human Rights
WHO: World Health Organization
